# Resilience of the primary health care system – German primary care practitioners’ perspectives during the early COVID-19 pandemic

**DOI:** 10.1186/s12875-022-01786-9

**Published:** 2022-08-11

**Authors:** Sandra Stengel, Catharina Roth, Amanda Breckner, Lara Cordes, Sophia Weber, Charlotte Ullrich, Frank Peters-Klimm, Michel Wensing

**Affiliations:** Department of General Practice and Health Services Research, Heidelberg University Hospital, Heidelberg, Germany

**Keywords:** COVID-19 pandemic, Primary care, Primary health care, Resilience

## Abstract

**Background:**

Primary care is a relevant pillar in managing not only individual, but also societal medical crises. The COVID-19 pandemic has demanded a rapid response from primary care with interventions in the health care system. The aim of this paper was to explore the responses of primary care practitioners (PCP) during the early COVID-19 pandemic and to analyze these with a view on the resilience of the primary health care system from the PCPs perspective.

**Methods:**

Shortly after the first COVID-19 wave (July—October 2020) *n* = 39, semi-structured telephone interviews were conducted with PCP in practices and at Corona contact points (CCP) in Baden-Wuerttemberg (Germany). Qualitative content analysis was applied, and the evolved categories were related to in a framework for resilience.

**Results:**

Primary care had an overall strong ability to adapt and show resilience, albeit with wide variance in speed and scope of the responses. When *coping with uncertainty,* the reasons given by PCPs in favor of opening a CCP mainly involved intrinsic motivation and self-initiative; the reasons against doing so were i.e. the lack of personal protective equipment, problems with space, and worries about organizational burden. A strong association existed between the establishment of a CCP and the use of resources (i.e. existing networks, personal protective equipment, exercising an office of professional political function). Our study predominantly found *adaptive aspects* for measures taken at medical practices and *transformative aspects* for setting up outpatient infection centers. PCPs played an important role in the coordination process (i.e. actively transferring knowledge, integration in crisis management teams, inclusion in regional strategic efforts) reaching a high level in the dimensions *knowledge* and *legitimacy*. The dimension *interdependence* repeatedly came into focus (i.e. working with stakeholders to open CCP, interacting among different types of primary care facilities, intersectoral interfaces). A need for regional capacity planning was visible at the time of the interviews.

**Conclusions:**

The results can be used for practical and research-based institutional and capacity planning, for developing resilience in primary care and for augmentation by perspectives from other stakeholders in the primary health care system.

**Supplementary Information:**

The online version contains supplementary material available at 10.1186/s12875-022-01786-9.

## Background

One and a half years after the beginning of the COVID-19 pandemic, health care systems around the world are still facing long-term challenges. Differing rates of infection and case mortality are being described worldwide and within Europe [1, 2]. Germany has recorded 3.8 million COVID-19 infections and over 90,000 deaths in the middle of the German fourth wave of COVID-19 with the Variant of concern Delta [3, 4]. Relating to data from the plateau of summer 2020 and the second wave of COVID-19 Germany had fewer COVID-19 deaths than the European average whereas when compared internationally with other high-income countries, it ranges in the middle [1, 4, 5]. Primary care is a part of the health care system that is a relevant pillar in the management of medical crises, including the current COVID-19 pandemic [6–10]. The COVID-19 pandemic has demanded a rapid response from primary care with interventions in the health care system that are the subject of diverse qualitative and quantitative studies across the globe [7, 11–16].

Germany has a decentralized ambulatory health care system that is mandated by federal and non-uniform state pandemic plans to provide medical care to the extent possible during pandemics [17–19]. Baden-Wuerttemberg is the third largest federal state in Germany where over 8,000 primary care practitioners in private primary care practices (PCP) form the basis of primary health care. In this setting, the response to the challenges in the early phase of the COVID-19 pandemic involved a restructuring of primary care and, at the same time, the creation of new “Corona contact points” as a form of a supplementary system of primary outpatient care [20, 21] (Table 1).

**Table 1:** Overview of Corona contact points as supplementary system of primary outpatient care

Name	Description
COVID-19-specialized primary care practices (CSP)	These were set up in parallel to regular primary care to treat patients with (suspected) COVID-19 and integrated into existing primary care practices
Outpatient infection centers (OIC)	These offer care for the same patients with (suspected) COVID-19 at independent facilities external to regular medical practices
Swabbing points	These offer exclusively the measure of taking a swab without examination or treatment of a patient and can supplement an outpatient infection center or be designed as a stand-alone-institution

These new Corona contact points fell under the supervision of the Association of Statutory Health Insurance Physicians (ASHIP) in Baden-Wuerttemberg. Under Sect. 72 of the German Social Code V, the Association of Statutory Health Insurance Physicians must ensure outpatient medical care for all persons with statutory health insurance [22].

The number of Corona contact points was subject to a period of rapidly changing dynamics: On 15 June 2020 there were 51 Outpatient infection centers (OIC), 204 COVID-19-specialized primary care practices (CSP), and 16 swabbing points in Baden-Wuerttemberg; on 22 October 2020, 15 OIC, 1124 CSP, and 36 swabbing points [23]. In total, over 90% of patients with (suspected) COVID-19 received ambulant care [24].

For the management of future crises, it is important to document and analyze the responses in previous crises [25–29]. In its COVID-19 Strategic Preparedness and Response (2021), the WHO lays out how to build resilience in health systems [30]. Turner et al. call for a broadening of the view at the organizational level of the health care system and for the application of a theoretical framework when conducting further studies on the COVID-19 pandemic response [31].

For this study the conceptual framework developed by Blanchet et al. was chosen, which specifies the domains in the management of resilience of health systems [32]. In this context “resilience” is defined as the “capacity of a health system to *absorb*, *adapt* and *transform* when exposed to a shock such as a pandemic, natural disaster or armed conflict and still retain the same control over its structure and functions”: During the absorption of the shock, the same level of resources and capacities are used as before; organizational adjustments are necessary during the adaptation, and functions and structures must be adapted during the transformation in order to respond adequately to the event. The capacities of the dimensions “knowledge”, “coping with uncertainties”, “interdependence”, and “legitimacy of institutions and norms” are taken into consideration [32]. Biddle et al. also identify this framework as a suitable lens through which to view the resilience of health systems, and moreover argue for an expansion of the framework [33]. Recently, Saulnier et al. have called attention to the need of further methodologically sound health systems research on resilience and to a shared bottom-up approach involving different stakeholders. They identify the need to record the developments and changes in health systems for the purpose of investigating the forces and dynamics [34].

The aim of this paper was to explore in a first step the responses, including related reasons as well as strategies of primary care practitioners during the early COVID-19 pandemic and in a second step to analyze these with a view on the resilience of the primary health care system from the perspectives of the primary care practitioners.

## Methods

### Study design

This exploratory qualitative study was conducted within the scope of the *PrimaryCovCare* research project as part of *Lessons learned—Studie MWK COVID-19*, a project undertaken by the medical schools in Baden-Wuerttemberg. The main objective of the observational study *PrimaryCovCare* is to document the experiences and perspectives of ambulant care models during the early phase of the COVID-19 pandemic in Baden-Wuerttemberg. The quantitative part has been published previously [20, 21]. The qualitative part described here was carried out after receiving a positive vote from the ethics committee with regard for COREQ (Supplement 1). To explore perspectives of primary care practitioners semi-structured telephone interviews were used for data-conduction and qualitative content analysis for data analysis.

### Recruiting and sampling

Recruiting was done from July to October, 2020, between the end of the first COVID-19 wave and the beginning of the second with the resulting heavy, pandemic-related workload in the target group. A random sampling strategy, followed by drawing a purposive sample from volunteers was targeted to reflect all types of facilities, locations, regions, and time point of opening. Recruitment was done among the primary care practices through several random drawings of n = 8,135 primary care physicians from 44 administrative districts in Baden-Wuerttemberg, who were identified via the ASHIP website. For the CSP group, recruitment was done by random name drawing identified via ASHIP. The medical practices were contacted by telephone and information was sent in case of interest. The slight willingness to participate resulted actually in participation of all volunteers. Because of bureaucratic hurdles, such as no availability of public contact information for the OIC, and insufficient response rate despite several drawings, a purposive sampling strategy was supplemented by a convenience one: emails were also sent to existing contacts known to the study team (SS). This strategy was also considered necessary for recruitment of the late opened CSP, which could not be identified via the ASHIP website. OIC management personnel were identified using the websites of physician associations and drawing on existing contacts from the study team; information was then sent via email (SS). If interest was indicated, more information was given by telephone and written consent was obtained. Participation in the interviews was voluntary and connected with the incentive of 50 euros.

### Data collection

The semi-structured interviews were held in German by telephone (AB, CR, LC, SS) and recorded using a digital audio-recording device. The sociodemographic data was collected by telephone before recording. During the interviews, pseudonymized notes were taken. The semi-structured interview guide in German for the PCP and Corona contact points were generated in a multidisciplinary group (five scientists: FPK, SS also primary care practitioner; AB, CR, MW (Public Health, Health Services Research and Implementation Science in Health Systems); LC (master’s degree candidate in Health Services Research and Implementation Science in Health Systems) on the basis of the research questions, the available literature, and seven orientation interviews. The interview guide compromised three topcis: a) early pandemic responses, b) continued operation of the facility in the coming months, and c) preparedness for future pandemics. Due to the difficult pandemic-related recruitment situation, the pilot interviews data were integrated into the study data, which was assessed as appropriate with regard to resulting minor revisions. Due to rapidly changing dynamics over the course of the pandemic, additions to the interview guide were made in September, after nearly half of the interviews had been held, to capture recent events (Supplement 2; Supplement 3). Qualitative data were collected up to the point of saturation as perceived by the research team.

### Data analysis

After transcribing and pseudonymizing interviews data analysis war performed according to qualitative content analysis [35]. SS and LC familiarized themselves with the data reading all transcripts and making case summaries. Data analysis then consisted of two steps: 1) Predominantly inductive categories and subdomains were formed based on the material in the explorative setting. Based on four independently coded interviews (CR, SS) categories and subdomains were defined. Next, 20 interviews were coded by two researchers each (LC and SS, or SW and SS). The results were divided into four main categories: a) *coping with uncertainty, b) adaptive and transformative measures, c) medical care* and d) *adaptations by the primary care physicians.* The data were processed, analyzed and coded by AB, CR (practical experience in qualitative research), SS (post-graduate training in qualitative research) and LC, SW (master’s degree candidate in Health Services Research and Implementation Science in Health Systems): Consensual coding showed an agreement of at least 90%, representing an acceptable intercoder reliability. The remaining interviews were coded by a single coder (SS, LC, SW) and checked by a second one. 2) During the coding process the high agreement with the conceptual framework for the dimensions of resilience governance (adapted from Lebel et al. [34] and described by Blanchet et al. [30]) was noted, so in the second step, the results for each main category were matched to the framework. The research process of data coding and analysis was continually discussed by the study team and supplemented through peer feedback in the internal departmental interdisciplinary research workshop (led by CU, sociologist, expert in qualitative methods). Data coding and analysis was supported by MAXQDA 2018 software. The first drafts of the manuscript were written in German and afterwards translated by a professional translator. Quality of the translation of the exemplar quotations were check for accuracy by the research team by translating them back to German.

## Results

### Description of the sample

A total of *n* = 39 interviews was held, of which one recording from an OIC was excluded from the analysis due to acoustic incomprehensibility. The interviews lasted on average 31.5 min (minimum 15.4; maximum 51.3 min). The characteristics of the sample are presented in Table 2.

**Table 2:** Characteristics of the interview sample (*n* = 38 interview participants)

	Regular primary care practices	COVID-19-specialized primary care practices	Outpatient infection centers
n (%)	13 (34.2)	14 (36.8)	11 (28.9)
primary care practitioner (%)	100	92.9^a^	100
Urban location (%)	53.8	21.4	54.5
Sex f (%)	38.5	28.6	45.5
Single practice (%)	46.2	21.4	
Age in years mean (SD)	53.2 (10.7)	51.5 (8.8)	52.4 (9.4)
Region in Baden-Wuerttemberg (%)			
North Baden	61.5	42.9	36.4
Opening (%)			
Early March 2020	-	35.7	45.5
Early April 2020	-	35.7	45.5
Late May – Sept. 2020	-	28.6	9.1

^a^one pediatrician included (special role of pediatrics in Germany with roles both in primary care and as specialty)

The first part of the results presents the response of the primary care practitioners and the explanation of the predominantly inductive developed main categories with subdomains.

### Coping with uncertainty

The situational starting point of the pandemic in March/April 2020 was described by the interviewees as a context of increasing COVID-19 case numbers, existing uncertainties associated with the expected, an awareness of scarce personal protective equipment in most primary care practices, and considerations regarding hospital capacities. A heterogeneous reaction was seen with differences in the decision for or against opening a Corona contact point (CSP or OIC) and the time point for such an opening.

#### Reasons for opening a Corona contact point

Given the context of uncertainty, most interviewees saw intrinsic motivation and self-initiative as the impetus behind the CSP and OIC that opened in early March and April.

*“And the initiative came relatively quickly and simply from us: we have to do something together with other active primary care practitioners.”* Interview 10 CSP, primary care practitioner

Extrinsic motivations were also identified: A few primary care practitioners reported one sole reason or an external mandate, for instance, from the regional administrative authority. The ASHIP was perceived by a few of the interviewees as being part of the decision to establish an early Corona contact point. Cited in connection with this was an active inquiry per email about potential volunteers to set up CSP early on in the pandemic and the suggestion of prioritized deliveries of personal protective equipment. If the decision in favor of opening a CSP was taken later, the reasons cited by interviewees were better compensation for providing the same care already being given to patients with (suspected) COVID-19 and promotion by the ASHIP and the Association of Primary care practitioners.

#### Reasons against opening a Corona contact point

The reasons against opening a Corona contact point cited by PCP and the CSP which opened later included organizational aspects such as the lack of personal protective equipment and location problems. Furthermore, PCP frequently expressed concerns about becoming swamped by external patients; other reasons were too much effort, lack of team consensus, lack of financial incentives, and reservations about the strategy.

*“Because essentially we were imagining or, to put it more precisely, afraid that we would find ourselves in over our heads with this.”* Interview 33, PCP, primary care practitioner

In the group of CSP that opened later on, the existence of other Corona contact points and a colleague in a risk group were cited.

#### Access to existing resources

Nearly all OIC and CSP reported using resources to implement the Corona contact point in the initial crisis situation. More than half of the interviewees reported having access to an existing network or current connections.

*“That I also have the responsibility of the emergency service in my area means that I have a good network of colleagues whom I can pretty much turn to.”* Interview 4 OIC, primary care practitioner

Around half had possibilities to access personal protective equipment. Mentioned several times was having an active role in a professional political function: local medical association, ASHIP (particularly those serving emergency service), professional associations. Access to staff and space to provide care to infectious patients was for several CSP a factor in opening. An implemented quality management system was described by two interviewees as a resource for pandemic management. The pandemic influenza plan, embedded within the practice’s quality management system after the swine flu pandemic 2009, was mentioned by only one person. Access to resources played a role for only a few interviewees in the PCP group: Besides personal protective equipment, an established, well-versed medical team was also mentioned.

### Adaptive and transformative measures

The measures implemented by the primary care physicians are presented based on the type of facility. The OIC were set up apart from PCP structures and are therefore presented separately. In terms of the adaptations in the setting of the primary care practices, there is a high degree of overlap and seamless transition between PCP and CSP so that these two are presented together.

#### Outpatient infection centers

The primary care physicians managing OIC had to establish entirely new practice structures. Relevant mentioned aspects in the setup were coordination, space, equipment and organization. An overview of all identified aspects regarding these organizational aspects is presented in Supplement 4.

Almost all OIC heads reported collaborating with other stakeholders. The range of inclusion was very heterogeneous spanning from joint planning with all relevant stakeholders from the beginning to a unilaterally initiated project rejected at first by the municipal administration. In some places partial work packages for opening were assumed by stakeholders who were not in primary care.

*“Then, I needed a tent and only the fire station has the tent, so then I said: ‘Good, then the fire brigade should set it up immediately so that we don’t find ourselves standing there on the weekend,’ And the tent was set up on Saturday.”* Interview 28 OIC, primary care practitioner

*„We had a bit difficulties to get a location from the local authority. That took a bit longer. About 2 weeks, until the mayor agreed.“* Interview 12 OIC, primary care practitioner

The ASHIP was named several times as an organizational guide, for instance, in terms of acquiring supplies, software installation, and for getting approval to operate. Staff organization was subject to OIC management, except in the case of one takeover by a physicians’ network. Finding staff was often through personal contacts. Furthermore, the use of existing network structures (the emergency medical services officer at the ASHIP, physicians’ network) was mentioned.

**Fig. 1:**
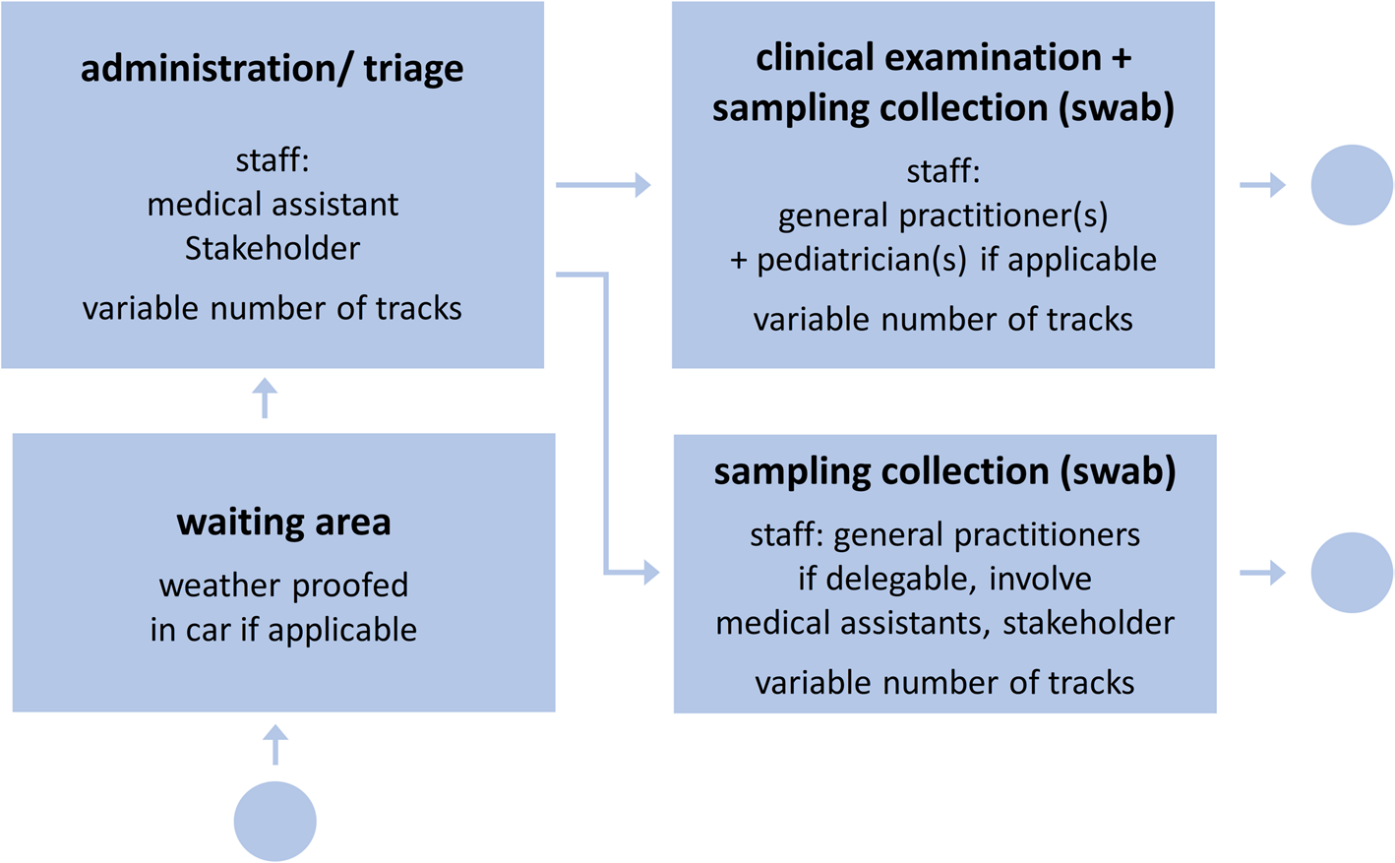
Structure of the outpatient infection centers. These aspects were taken from the responses of *n* = 11 outpatient infectious centers

The defined core task of the facilities ranged from serving only as a (temporary) swabbing station to a OIC meant to provide detailed diagnostics and triage for severely ill patients. A summary of the identified aspects regarding the structure of the non-uniform OIC is presented in Fig. 1.

In one example, a regional plan was laid down for five locations from the beginning with bundled tasks assigned according to the available resources. In all of the other cases, the planning that was described was limited to one location.

The reasons for a particularly early time point for opening and quick set up could not be specifically gleaned from the information. In the quickest cases, it was noticed that there was an expedient option to organize space; the absence of this criterion, however, did not conversely indicate no particular quickness in opening, so that a multifactorial genesis must be supposed in the analysis.

#### COVID-19-specialized primary care practices and regular primary care practices

The adaptations made in primary care practices were overall very heterogeneous, an aspect that was visible in the different attitudes toward opening a CSP or retaining a regular primary practice. In contrast to the OIC, only two of the CSP reported working with other local stakeholders. Management via telephone had a higher importance in both groups; video consultations were reported in only two cases and faced lower demand. The procedures after filtering by pone were heterogeneous and depended on the practice and the time point in the pandemic.

Measures to separate physical spaces were implemented differently. There were major structural changes to the building at several of the CSP, but only at a few PCP. Organizational measures for spatial separation and hygiene measures were, for the most part, described consistently. To separate infectious and non-infectious patients in terms of scheduling, some of the PCP and CSP held specific appointment times for infectious patients; a few of the CSP were even able to provide appointments in parallel for infectious and non-infectious patients. A portion of the PCP did not offer routine care to patients with (suspected) COVID-19 in their own practice spaces. The implementation of specific times for appointments with infectious patients was very heterogeneous. Only a few CSP made statements about quality management.

The patterns of use for the Corona contact points were also described in a very heterogeneous manner. Almost all of the CSP that opened early and half of those that opened later and only one PCP used no external contact points.

***“****No, we treated all of the patients ourselves.”* Interview 3 PCP, primary care practitioner

A heterogeneous situation regarding use was seen among the PCP ranging from one appointment at the beginning to partial and predominant use.

*“(...) referral of adults to the COVID-19-specialized practices or the outpatient infection centers (...) when it is necessary. We speak with the patients and refer them only if we suspect that there is a risk of infection.”* Interview 23 PCP, primary care practitioner

None of the CSP made continual use of an external contact point. A summary of all of the identified aspects for organizing the adaptations in the primary care practices is presented in Supplement 5.

### Medical care

#### Patients with (suspected) COVID-19

The range of experience with patients with (suspected) COVID-19 differed at the time of the survey spanning from no confirmed cases by swab^1^ (PCP) to 100 confirmed cases by swab (CSP) at that time. Even SARS-CoV-2 positive cases in the own practice not necessarily led to an examination and direct contact with these patients, for example if they were asymptomatic and the test was conducted at a Corona contact point:

*“So (...) we have not yet had to examine COVID patients because the two who tested positive did not actually have COVID. They just tested positive.”* Interview 39 PCP, primary care practitioner

The care of patients with (suspected) COVID-19 was described heterogeneously by the group and ranged from one prevention of a patient entering the practice through telephone filtering and referral to a Corona contact point to comprehensive care including a SARS-CoV-2 swab inside the practice space. The extent of the physical examination was, as a result, very heterogeneous, which according to one interviewee led to delayed diagnosis of a severe non-COVID febrile infection.

*“Few of those with fever actually have corona. So, we have to see exactly these patients. It can also end up as something quite different, yes.“* Interview 11 CSP, primary care practitioner

*„I have referred to patients, who came with an infection of unknown cause. These, we couldn´t care them adequately in the beginning, nobody could this.”* Interview 11 CSP, primary care practitioner

One CSP reported carrying out more diagnostics on external patients. The care in nursing homes was not a topic for most of those surveyed. Some CSP and OIC reported taking on the care of patients with (suspected) COVID-19 in nursing homes or models for agreements regarding resource-based care of such patients. Making home visits to patients with (suspected) COVID-19 were reported only by a few. Only one CSP reported monitoring patients with (suspected) COVID-19 by telephone. In three interviews pediatric care was clearly identified as a special aspect based on the need for special equipment and a higher importance of treating young patients in familiar, trusted surroundings than was the case for adults. The topic of pediatric care was not raised in most of the interviews.

#### Patients without (suspected) COVID-19

At the beginning of the pandemic there were consistent reports of a reduction in care, including home visits, and a switch to mainly providing care by telephone, especially for routine examinations.

*“So, I no longer feel that people are frightened to come into the medical practice now. That was *very*, *very *strong at the beginning.”* Interview 18 CSP, primary care practitioner

A portion of the PCP reported that patients with an increased risk for COVID-19 were only examined in the practice if they tested negative for the virus. Some of the PCP reported delayed routine interventions in hospitals and at specialized clinics, which was not classified as a shortage of care. Two PCP reported delayed hospital admissions due to the failure to admit patients with acute disease and that did result in insufficient medical care. Some of those surveyed reported an initial fear of contagion on the part of patients who then did not come to the practice. One PCP and two CSP concluded from this that diagnoses were made later and treatment of acute conditions started later resulting in unmet medical needs.

### Adaptations by the primary care physicians

#### Primary care physicians in the process of coordination

Improved collaboration with public health authorities, hospitals, nursing homes and the statutory health insurance providers was reported especially by CSP but also by some OIC and PCP as a result of getting to know each other personally and exchanging ideas and information. Several of the OIC and almost all of the CSP reported performing mass swabbing during outbreaks. In this process, CSP and one PCP were also partially involved in giving medical care; the OIC only took swabbed samples. Half of the OIC and a several CSP took on an active role in transferring knowledge. Several OIC heads and one CSP head were integrated into crisis management teams. A percentage of those in lead management positions at CSP and OIC reported taking on coordinated, regional strategic efforts and/or being involved in them. One OIC head reported on the setup of a new regional office for the ASHIP pandemic officer who was officially appointed in August 2020, something which can be viewed as an official legitimization of the coordination efforts that had already been undertaken.

*“At some point, ... said that I can appear publicly as the official representative of the ASHIP for [PLACE] and so I also became the pandemic officer for [PLACE] a couple of weeks ago.”* Interview 31 OIC, primary care practitioner

Several OIC reported an expansion or deepening of the exchanges in the regional networks, in two cases with cross-regional sharing.

#### Communication and sharing of knowledge

This topic was present in all of the groups. Unilateral utilization of information was primarily described in the PCP group as needing to be actively gathered (e.g., Robert Koch Institute, newspapers, German College of General Practitioners and Family Physicians, ASHIP website, public health authorities), but newsletters were also mentioned (e.g., ASHIP and the medical association). The OIC and CSP described, above all, the development of active channels for information to medical colleagues in the region, for example, via mass emails, newsletters, and WhatsApp groups. One interviewee drafted an OIC structure template for the entire administrative district. Communication as the means to transfer knowledge showed itself primarily in the OIC and CSP groups. “Small networks” played a role here as a way to exchange information with others. Special developments included active feedback by three interviewees from the circle of regional colleagues in higher-level structures and the holding of a post-graduate training course.

*“So, all (…) who were there [crisis management team] (…) were asked to report on the current situation and I (…) used that to bring up the problems faced by locally practicing primary care physicians.”* Interview 28 OIC, primary care practitioner

Communication took place in the team at the practice level, in the team between OIC, in WhatsApp groups, and also by learning about the experiences of others.

#### Evaluation of the Corona contact points

A large degree of heterogeneity was visible among the OIC in regard to level of utilization. Some of the OIC were closed at the time of the survey for lack of demand. A repurposing to serve as swabbing points was described in part; other OIC were open. Acceptance was described differently. The reasons cited for a high level of acceptance were many collaborating colleagues and regional, long-established circles of colleagues. A good level of demand was predominantly described in the CSP group through referrals by colleagues and often by the public health authorities. One PCP performed individual patient swabs for colleagues with a solid relationship of trust.

Preventing an underuse of medical care in PCP and patients who were not patients of a specific primary care physician were named as the indications for Corona contact points. For many, the OIC made sense only if case numbers increased or if they were located in urban centers. Swabbing points appeared to mainly be perceived as relieving other medical care facilities. A portion of the surveyed PCP expressed reservations in theory about CSP, for instance, unjust compensation.

[Regarding CSP] *“Bad, really bad. Because I do my job on my own for my patients, because they are from some village ... because if they receive money for that, ... then they should also do the work.”* Interview 21 PCP, primary care practitioner

#### Capacities of the primary health care system

Over the duration, several CSP and OIC perceived that more PCP were performing their own swabs and that more CSP existed. Many CSP and PCP reported a return to normal operation at the time of the survey and the availability of a sufficient supply of personal protective equipment. Almost all of the PCP and CSP reported ideas about expanding capacity, but without a defined concept for about half of the PCP and some of the CSP. In groups of CSP it was possible to see a higher level of institutionalization of the consultations with infectious patients, correlating with a tendency for more detailed advance planning. One CSP called attention to the need for regional coordination. Different concrete ideas were named by some OICs with preparations for reopening or expanding. In the case of three there was an inclusion of a regional step-wise plan, but only once were concrete reasons for its activation mentioned.

*„(…) once, the [special profession] was present in spring, who has asked, what are you doing, if 1000 sick people would attend (…).We made a plan to which town hall we would go”.* Interview 17 OIC, primary care practitioner

*„Well, we already have agreed on a stepwise procedure in the district. The biggest outpatient infection center in the center of our district can be ramped up within 48 hours. Another one within 48 hours, and we assume, that 3 centers will be sufficient.”* Interview 4 OIC, primary care practitioner

Some mentioned the errors of such plans despite recognizing their potential relevance. The ASHIP was named in two interviews as the guiding body for the OIC, without a clearly recognizable strategy for doing so. Some interviewees cited actual numbers that they themselves were not able to exceed. The identified factors influencing health care capacities are listed in Table 3.

**Table 3:** Factors influencing infectious patient capacity

**Specialized and regular general practices**	**Outpatient infection centers**
- Working hours/ opening hours ↑ ↓ - Pulsing frequency of infectious consultations - Treatment by phone or video - Planning of spatial capacities - Planning of personnel step-up or back-up - Use of specialized contact points ↑ ↓	- Opening hours ↑ ↓ - Number of tracks ↑ ↓ - Repurposing tracks/ facility (i.e. sample collection only versus offering examination) - Pulsing increase through optimization of procedures - Relocation - Staff: hold available; include stakeholders - Preparedness (re-) opening
Necessity of a forward-looking regional plan with phases	
** Limitations**	
- Personal limits - Spatial capacities - Human resource capacities - Quality ↓ when capacity limits are exceeded at the facility	

These factors were taken from the responses of *n* = 27 specialized and regular general practices and *n* = 11 outpatient infection centers

Table 4 presents the second part of the results: the matching between the described response and the conceptual framework on resilience [32].

**Table 4:** Resilience of the primary health care system from primary care practitioners´ perspectives

**Response—Main categories**	**Matching to the conceptual framework on resilience by Blanchet et al.**^a^
**Coping with uncertainty**	The results of the analysis were directly assigned to the dimension **coping with uncertainty**: a heterogeneity of the response became apparent. Especially intrinsic motivation and self-initiative, but also resources, such as access to personal protective equipment, staff and space, fostered the opening of a Corona contact point. Furthermore, **interdependence** was seen, whether it be through community representatives or the Association of Statutory Health Insurance Physicians (ASHIP), in the external influence on the motivation that played a more significant role in the later decisions to open a Corona contact point. Moreover, connectedness to existing networks and an active role in professional political functions had an accelerating or amplifying effect here on the pandemic response
**Adaptive and transformative measures**	Opening an OIC was placed under **transformative capacities** because this involved not only the creation of new structures, but also required taking on the management of a new type of center with new patient pathways. The dimension **interdependence** also played a major role in the coordination among the stakeholders during the set-up process. On the one hand, a high degree of capacity regarding **legitimacy** was seen because the role of the primary care providers was accepted; on the other, the assignment of roles and leadership positions was often not clearly defined. Generally, the changes to the PCP and CSP could be mainly categorized under **adaptive capacity**, since they consisted primarily of organizational adaptations, while the function and structure remained unchanged. The use of Corona contact points and the major alterations to buildings that were seen at some CSP and individual PCP are classified as processes of **transformative capacity**. Overall, the results pointed not only to a high level of adaptability and resilience of primary care, but also to a high degree of heterogeneity in both speed and scope of the crisis response
**Medical Care**	Above all, aspects of the dimension **interdependence** became clear. These were seen, for instance, in how patients behaved, at the interface between ambulatory specialists and the hospital sector, within primary care through the use of Corona contact points, and the division of medical care in nursing homes. **Transformative aspects** can also be derived from the holding of mainly remote telephone consultations. **Adaptions** to maintain the quality of medical care succeeded in varying levels
**Adaptations by the primary care physicians**	Aspects of the dimension **interdependence** were topics in the collaboration with different stakeholders, such as public health authorities, hospitals, nursing homes, the ASHIP, and other primary care providers in the use of the Corona contact points, and a developmental process was visible. In particular, the CSP and OIC played a decisive role in the transfer of **knowledge** and often took over an active role with the integration of **transformative processes**, such as the formation of communication groups or giving feedback from the field of primary care, while at the same time a great amount of heterogeneity was seen in the behavior of those surveyed. The inclusion of primary care physicians in the coordination process by means of integration into crisis management teams, taking over regional coordination tasks, and, finally, establishing an official office for the pandemic officer appointed by the ASHIP reflect a high degree of resilience with regard to the dimension **legitimacy**; however, there were also gaps in the regional inclusion of primary care in the initial pandemic situation

^a^The framework includes the capacities of a health system to absorb, adapt and transform during a crisis and the dimensions “knowledge”, “coping with uncertainties”, “interdependence”, and “legitimacy of institutions and norms” [32]

## Discussion

Our main findings point not only to a high level of adaptability and resilience of primary care overall, but also to a high degree of heterogeneity in both speed and scope of the crisis response.

Internationally, rapid adaptations in primary care as a response to the COVID-19 pandemic were described in many instances around the world with measures to reduce contacts through telephone consultations in general practices and the creation of primary care centers external to regular medical practices [7, 8, 12, 36]. As a consequence of the *adaptive and transformative measures* taken by the primary care practices described in this paper, it was possible from the very beginning to decentrally include some of these practices in the active, in-person care of COVID patients, in that they set up COVID-19-specialized practices or held such appointments as part of their regular practice. In addition, independent external Corona contact points were also set up quickly in the form of OIC. Compared to reported mediocre results in the assessment of primary care access [37], the German primary care system seems to be more strongly positioned in the ongoing crisis.

When *coping with uncertainty*, the main reasons for opening a Corona contact point early on were intrinsic motivation and self-initiative; the reasons against such a move were, above all, the lack of personal protective equipment, problems with space, concerns about organizational strain, the absence of financial incentive, and hesitations about the conceptual strategy. In regard to the lack of preparedness, there is a high consistency in the literature about the initial absence of personal protective equipment [13, 38, 39]. Taking the initiative and the importance of networks are also visible in other German papers [16, 40]. There is a strong correlation between setting up a Corona contact point and using resources such as existing networks, personal protective equipment or exercising an office of professional political function. Above all, COVID-19-specialized primary care practices demonstrated a high degree of organization and more detailed advance planning compared to the regular general practices. Indications of differences in the responses of different general practices are also seen in Danhieux et al. [41].

According to our knowledge, this is the first systematic and comprehensive description of the set up and organization of differently structured OIC in Germany. What is noticeable in the results is that there was no mention of strategies upon which could be drawn. The *interdependence* that was also seen among stakeholders and within primary care could supplement German data relating purely to general medical practices [16, 40] and link to reports from China and South Korea, where the early establishment and structure of drive-throughs and OIC-like centers are described [42, 43]. Differences between the German system and the infection clinic described by Jiang et al. are mainly seen in their size and placement in the hospital setting [42]. In terms of the organizational approaches in general practices, a high level of conformity was seen in the German literature, for example, when looking at measures implemented to separate patients or the protective measures. The establishment of a CSP system was not mentioned in other papers, but it is impossible to say if this is due to different federal structures [44] or a lack of data [16, 40].

The dimension *interdependence* came repeatedly to the forefront, for instance, when working with stakeholders to set up Corona contact points, the interplay between different primary care facilities, hesitations about the idea of CSP, when considering patterns of patient utilization or the intersectoral interfaces, and was subject to an ongoing developmental process. A positive correlation was also seen here to existing networks, and very clearly visible were the supportive effects on these networks. In terms of triangulation, the role of the ASHIP in setting up the Corona contact points showed itself to be more pronounced in the survey conducted by us in Baden-Wuerttemberg [20]. When looking specifically at the relevance of the stakeholders, the necessity of including them at the organizational level becomes clear [31, 34], and across states the importance of inter-organizational collaboration to manage the COVID-19 pandemic is confirmed [8, 15, 16, 31].

In regard to health care, it became very clear, given the backdrop of a real risk of a shortage of medical care and very heterogeneous processes, that the quality of care for infectious patients and non-infectious patients must be targeted during a pandemic and, furthermore, special needs regarding home residents, domestic situations and children must be taken into account, which is also corroborated in the literature [12, 45–47]. This aspect is not explicitly anchored in the framework put forth by Blanchet et al. The high utilization of telephone consultations in comparison to video consultations is also confirmed in other papers published in Germany [48]. Evaluations of these strategies have yet to be undertaken.

Primary care physicians played an important role in the coordination process, for example, through active participation in knowledge transfer, integration into crisis management teams, appointment of the new ASHIP pandemic officer, or inclusion in regional strategic efforts, so that a high level of resiliency was also seen in the dimensions *knowledge* and *legitimacy*, a resiliency that can still be expanded further despite the heterogeneity and, in part, initially delayed responses. The need to include primary care physicians in coordinating processes is a topic that is also covered internationally [6, 10, 31]. The relevance of information and communication channels in the context of a pandemic is being reported on internationally [6, 10, 26, 31].

At the time of our interviews, the need for capacity planning that is forward-looking, regional, and spans the different types of medical facilities became visible when considering the identified *interdependence*, whereby questions about responsibility and leadership remained open.

Rawaf et al. show the necessity of ensuring access to primary care for all medical reasons during the pandemic [7], which appears to have been successfully achieved in the context examined here with a heterogeneous decentralized system and local adaptations. This also includes offering services for different needs in the urban and rural settings [49]. In summary, primary care physicians contribute substantially to crisis management. This demonstrated a high but further expandable resilience of primary care in Baden-Wuerttemberg during the early phase of the COVID-19 pandemic.

### Implications for practice and research

The descriptions of the response and resilience from the statements of the primary care physicians could be linked with other building blocks in research or practice with the goal of obtaining an overall view of the resilience of the primary health care system and potential steps for further development. This is successful particularly because the participating stakeholders were identified and the framework of Blanchet et al. was applied, which is explicitly designed for the use of different target groups [32]. Further research or practical approaches could link to the identified aspects regarding capacities or the development of future-oriented regional strategies and quality of medical care, and thus be used as approaches to increase resilience. However, the heterogeneity in primary care response has to be considered and is a challenge for healthcare planning. To anchor the quality of care for infectious and non-infectious patients as well as groups with special needs (i.e. children, home residents, home visits) in a framework of resilience could be discussed. The aspects for setup of Corona contact points and factors with influences on capacity, which are bottom-up developed, could serve as a tool-box for crisis management and orientation on best practice.

### Strengths and limitations

This study represents, to the best of our knowledge, the first qualitative survey of primary care physicians in Germany with systematic qualitative documentation of the response of primary care physicians in the COVID-19 pandemic. This study has an explorative approach, is limited to the statements made by primary care physicians in Baden-Wuerttemberg, Germany, but distinguishes itself by covering different types of facilities at which primary care is provided, inter-regionality, and by including different urban and rural districts. Generalizations or transferability to other German states or health care systems is not directly possible due to the study design and the heterogeneity [44, 50]. However, given the context of the methodology and the responses to the pandemic that have been similarly described around the world, it is possible to derive suggestions and make comparisons. A bias cannot be ruled out: It is possible that motivated physicians were more likely to have volunteered to participate which would mean there is an even higher degree of heterogeneity present in the response.

## Conclusions

The results can be used for facility and capacity planning in the outpatient sector and for ongoing preparation for crisis preparedness on the part of primary care. Such approaches could include maintaining and developing regional networks, integrating active members of professional political functions, defining responsibilities in a crisis, stockpiling protective supplies, planning for spatial capacities and organizational support for general practices in terms of crisis preparedness, and learning from each other. Self-initiative and intrinsic motivation on the part of primary physicians must be retained as a major pillar. Moreover, a bottom-up perspective should be augmented by including additional stakeholders of the primary health care system, the results should be discussed, and shared goals should be defined to strengthen resilience and pandemic plans. An intersectoral linkage in crisis planning [51, 52], and thus broaden the perspective beyond primary care appears valuable.

## Supplementary Information

**Additional file 1: Suppl.1.** COREQ-Checklist.

**Additional file 2: Suppl.2.** Interview guidelineCorona contact points.

**Additional file 3: Suppl.3.** Interview guidelineprimary care practices.

**Additional file 4: Suppl.4.** Organizationalaspects in the setup of outpatient infection centers.

**Additional file 5: Suppl.5.**  Aspects of adaptions in COVID-19-specialized and regular primary care practices in the beginning of the COVID-19 pandemic.

## Data Availability

The datasets generated and analysed during the current study are not publicly available due to European provisions for data protection, of which the interviewees were assured, but are available from the corresponding author on reasonable request.

## Abbreviations

CSP: Heads of COVID-19-specialized primary care practices; COVID-19-specialized primary care practices
PCP: Heads of regular primary care practices; regular primary care practices (as opposed to primary care practices that were repurposed to function as COVID-19-specialized primary care practices)
OIC: Heads of outpatient infection centers; outpatient infection centers
ASHIP: Association of Statutory Health Insurance Physicians in Baden-Wuerttemberg

## Footnotes

1 At the time of the interviews the confirmation of an infection with SARS-CoV-2 was conducted by PCR (polymerase chain reaction) of a nasopharyngeal swab.
