# Supplementary material for: Resilience of the primary health care system – German primary care practitioners’ perspectives during the early COVID-19 pandemic
Source: BMC Prim Care. 2022 Aug 11;23:203. doi: 10.1186/s12875-022-01786-9 (PMC9365682; doi:10.1186/s12875-022-01786-9)
Supplement: Supplementary file 2 — Additional file 2: Suppl.2. Interview guidelineCorona contact points. [file 12875_2022_1786_MOESM2_ESM.docx]

**PrimaryCovCare**

Health care models and processes for outpatients during the SARS-CoV-2 pandemic

Structured interview questions for the staff at the Corona contact points who participated in the PrimaryCovCare study

| **Structured question** | **Follow-up questions/ Aspects** |
| --- | --- |
| 1. Let’s go back and start by looking at the first phase of the pandemic. Can you tell us how it came about that you set up the COVID-19-specialized primary practice/outpatient infection center? | - What was the triggering event? - Who gave the order? Who worked with you on it? How much effort did it take? - Why was this contact point needed? - Were you prepared? - What made your practice suitable? |
| For new CSP that opened in July or later:  Let’s go back and start by looking at the first phase of the pandemic. Which measures/restructuring did you undertake to provide health care for your patients during the SARS-CoV-2 pandemic? * | - Did you treat patients with confirmed COVID-19 infections or did you refer them to a Corona contact point? - Why did you decide at first not to become a COVID-19-specialized primary care practice? - Why did you change your mind/approach as things progressed? - What made your practice suitable? * |

| 1. The pandemic has been marked in part by quickly changing dynamics, for instance, there can be a rapid increase in infections requiring differential diagnosis, a requirement to take swabs from returning travelers*, an outbreak of COVID cases that then quickly fades away. Given this situation, how do you envision the operation of your contact point in the coming months? |  |
| --- | --- |
| 2a. What challenges/obstacles do you see in terms of providing health care at your Corona contact point?  Or: Was there anything that you found especially helpful or problematic with… | - Are there particular challenges with organization? - Are there particular challenges with the general conditions? - What obstacles do you see regarding collaboration, interface management and coordination? - What challenges do you see regarding medical care for patients? - What obstacles do you see regarding use/acceptance? - How do you handle suspected cases that are only recognized later as suspected cases? - How is the interaction with nursing homes? |
| 2b. In your opinion, what helps to successfully provide medical care at your Corona contact point? | - In your opinion, what makes good organization possible? - What general conditions do you think are helpful? - What is conducive to effective cooperation, interface management and coordination? - What helps to provide medical care to those needing it? - What could promote the use/acceptance of your medical facility? |
| 2c. If your Corona contact point goes into standby or if the demand suddenly changes while it is in operation, what do you have to do to ramp up operations? |  |
| 2d. The number of Corona contact points has changed markedly from 200 CSP to 800 CSP. In contrast, the outpatient infection centers have been reduced from 51 to currently 8. What is your take on this development? * |  |
| 2e. The topic of vaccination is becoming more and more pertinent in this pandemic. In addition to the necessary vaccines against influenza and pneumococcal infections, the first vaccine against COVID-19 could soon be available. What do you think is important? * | (If this topic has not already been mentioned under 2.) |

| 1. You have gathered a lot of experience in this pandemic. We need to assume that there could be other pandemics in the future. In your opinion, what should be done so that the ambulant sector is prepared in the future? |  |
| --- | --- |
| 3a. What challenges/barriers must be overcome? | - What general conditions would be important? - What structures are suitable? - How do you view interpandemic preparation? - Who should assume the leadership/management/coordination? - How do you envision your role in making decisions/exerting influence? - What would an appropriate flow of information or communication look like? - Which stakeholders would need to be integrated into such communication? |
| 3b. What would contribute to successful management of a future pandemic? | See 2b. |

“We’ve come to the end of the interview. Would you like to add anything about this topic?”

“Thank you very much for participating in the PrimaryCovCare project and agreeing to be interviewed. Thank you for taking the time!”

***** These texts were added in the middle of September due to the rapidly changing situation in the ambulant sector and serve to focus the questions more sharply.
