# Supplementary material for: Resilience of the primary health care system – German primary care practitioners’ perspectives during the early COVID-19 pandemic
Source: BMC Prim Care. 2022 Aug 11;23:203. doi: 10.1186/s12875-022-01786-9 (PMC9365682; doi:10.1186/s12875-022-01786-9)
Supplement: Supplementary file 4 — Additional file 4: Suppl.4. Organizationalaspects in the setup of outpatient infection centers. [file 12875_2022_1786_MOESM4_ESM.docx]

Supplement 4 – Organizational aspects in the setup of outpatient infection centers

| Coordination | Stakeholder   - Primary care practitioners (often function in local medical association, Association of Statutory Health Insurance Physicians, or professional politics) - Association of Statutory Health Insurance Physicians - Clinics - Local government, includes district office with public health department, public safety authorities and organizations, mayor - Medical services - Local physicians’ network - Local sponsors   Other aspects   - Definition of goals - Distribution of tasks, responsibilities and leadership - Medical leadership: primary care practitioner |
| --- | --- |
| Space | - Transportation/arrival - Parking - Separation of infectious / non-infectious / population 🡪 consideration of situation, separate entrance/exit, number of rooms - Practicability of hygienic measures - Telephone, fax, Internet connection - Air-conditioning option - Duration of use - Sanitary facilities - Mobile (tent, module) versus regular facility - Consider suitability for drive-in |
| Equipment | - Personal protective equipment - Examination tables, medical equipment - Insurance Card reader - Electronic data processing (PC, printer) - Software (most-widely known as possible) - Vehicles for providing care to immobile patients / mass outbreaks |
| Organization | - Formal status (decision, if unique practice approval number) - Employee work schedule (consider availability, staff, tool) - Staff remuneration - Assumption of costs - Invoicing   Quality management   - Hygienic measures - Staff instruction - Responsibilities / delegation (consider leading medical assistant) - Patient pathways (consider making appointments, on-site procedures, results, lab) - Structured documentation - Knowledge transfer - Medical decision-making aids |

The aspects were taken from the responses by n = 11 outpatient infectious centers.
