# Supplementary material for: Resilience of the primary health care system – German primary care practitioners’ perspectives during the early COVID-19 pandemic
Source: BMC Prim Care. 2022 Aug 11;23:203. doi: 10.1186/s12875-022-01786-9 (PMC9365682; doi:10.1186/s12875-022-01786-9)
Supplement: Supplementary file 5 — Additional file 5: Suppl.5. Aspects of adaptions in COVID-19-specialized and regular primary care practices in the beginning of the COVID-19 pandemic. [file 12875_2022_1786_MOESM5_ESM.docx]

Supplement 5 – Aspects of adaptions in COVID-19-specialized and regular primary care practices in the beginning of the COVID-19 pandemic

| Cooperation | - Cooperation with stakeholders |
| --- | --- |
| Protective measures | - Personal protective equipment - General hygiene measures   Patient measures   - Instruction on using masks / hygienic hand disinfection - Information on entrance door; practice; phone; press; homepage - Sanctions (domestic authority)   Organizational measures   - Installation of a Plexiglas window at the reception - Markings on the floor |
| Handling by phone | - Filtering infectious / non-infectious patients - Treatment of patients by phone only - Option to obtain certificate of incapacity by phone - Monitoring infectious patients |
| Video consultation | - Often discussed but scarcely used |
| Team | - Team meeting for information and decisions - Responsibilities / delegation / division of work - Shared decisions versus chief decision |
| Quality management | - Integration of the pandemic plan into the practice quality management - Use of IT-based quality management information system - Development of questionnaires - Written updates for staff |
| Use of specialized contact points | - Reduce or prevent infectious patients inside the practice |
| Spatial separation | Structural adaptions   - Use of garage, garden house - Setup of a tent - Alteration of the building (i.e. basement room) - Use of space outside practice (i.e. module)   Organizational adaptions   - Filtering by phone - Filtering at the entrance - Changing the function of a consulting room to an infection room - Limitation of patients in the practice - Reduction of inside waiting space - Swabs through the window or outside |
| Organization of infectious consultation hours | - Equipment for medical examinations - Storage of personal protective equipment - Division of work, i.e. shifts, teams, staff reduction (direct contact) - Delegation - Organization of laboratory pick-up times - Hygienic measures - Patient pathways |

These aspects were taken from the responses of n = 13 general practices and n = 14 COVID-19 specialized primary care practices.
